# Automated landmarking of bends in vascular structures: a comparative study with application to the internal carotid artery

**DOI:** 10.1186/s12938-021-00957-6

**Published:** 2021-11-27

**Authors:** Henrik A Kjeldsberg, Aslak W Bergersen, Kristian Valen-Sendstad

**Affiliations:** Department of Computational Physiology, Simula Research Laboratory AS, Kristian Augusts gate 23, 0164 Oslo, Norway

**Keywords:** Geometric characterization, Automated landmarking, Internal carotid artery, Computational geometry, Landmark detection, Geometric risk factor

## Abstract

Automated tools for landmarking the internal carotid artery (ICA) bends have the potential for efficient and objective medical image-based morphometric analysis. The two existing algorithms rely on numerical approximations of curvature and torsion of the centerline. However, input parameters, original source code, comparability, and robustness of the algorithms remain unknown. To address the former two, we have re-implemented the algorithms, followed by sensitivity analyses. Of the input parameters, the centerline smoothing had the least impact resulting in 6–7 bends, which is anatomically realistic. In contrast, centerline resolution showed to completely over- and underestimated the number of bends varying from 3 to 33. Applying the algorithms to the same cohort revealed a variability that makes comparison of results between previous studies questionable. Assessment of robustness revealed how one algorithm is vulnerable to model smoothness and noise, but conceptually independent of application. In contrast, the other algorithm is robust and consistent, but with limited general applicability. In conclusion, both algorithms are equally valid albeit they produce vastly different results. We have provided a well-documented open-source implementation of the algorithms. Finally, we have successfully performed this study on the ICA, but application to other vascular regions should be performed with caution.

## Introduction

Stroke is the second leading cause of death, accounting for 11% of all deaths worldwide in 2015, and the main cause of disabilities [1, 2]. Two common causes of stroke are intracranial aneurysms, and carotid stenosis, both commonly harbored in the internal carotid artery (ICA), a tortuous vessel supplying blood to the brain [3, 4]. Specific morphological characteristics have been linked with vascular pathologies, leading to the concept of *geometric risk factors*, as introduced by Friedman et al. [5]. Consequently, correlating geometric features and pathologies in the carotid artery has been sought extensively [6–12]. However, the current analyses are often manual, and therefore operator-dependent and labor-intensive. Besides, studies are often conducted by non-experts within neuroradiology, which could cause differences in classification, and potentially influence the results [13–17]. Hence, a topic of interest is the development of a computationally inexpensive tool for automated and objective characterization of the ICA, referred to as landmarking. Previous methods have been presented by Piccinelli et al. [12] and Bogunović et al. [18], which rely on numerical approximations of the curvature and torsion, used to subdivide the vessel into separate bends, and are allegedly applicable to arbitrary tubular structures.

**Fig. 1:**
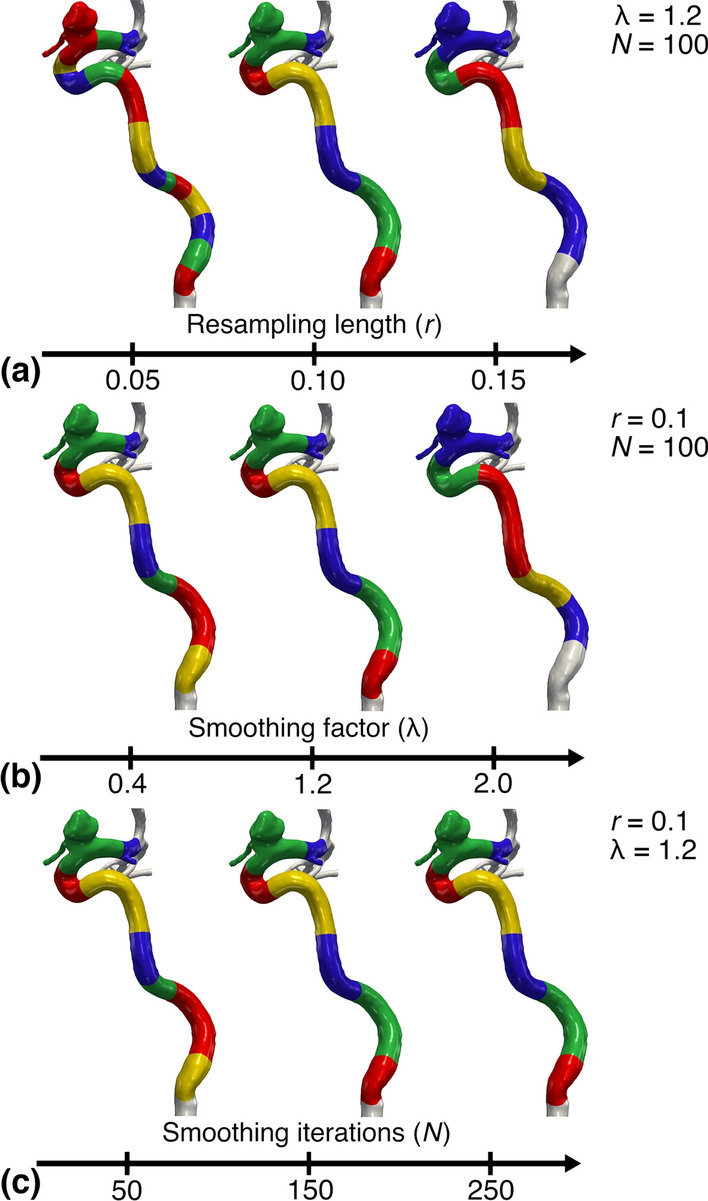
Results using the algorithm by Piccinelli et al., where we have varied the three main input parameters. For row **a**,**b**, and **c** we have varied *r*, $\lambda$, and *N*, respectively, for a representative model

However, to the authors’ knowledge, there are no open source-implementations of the algorithms, and information about the input parameters for estimation of the geometric properties are unknown, limiting their applicability. Furthermore, the algorithms capability of capturing the underlying morphology irrespective of segmentation variability remains unknown. Therefore, our goal is to investigate the existing methods for automated characterization of the ICA, which will allow for objective, consistent, and operator-independent medical image-based morphometric analysis.

We will revisit automated landmarking, and organize the remainder of this paper as follows: we devote Sect. to the results, consists of three separate subsections. More precisely, in Sect. , we investigate the sensitivity of the algorithms to input parameters for estimation of geometric properties, in the pursuit of a set of parameters that produce consistent landmarking results. In Sect. , we perform verification of our implementations, and compare the results of the two algorithms. In Sect. , we test robustness of the algorithms. We present a discussion for the three subsections in Sect. , followed by limitations and implications. Concluding remarks are presented in Sect. , followed by our methodology in Sect. , including computation of the centerline, the relevant geometric properties, and a presentation of the two existing landmarking algorithms.

## Results

### Sensitivity analysis

#### Piccinelli’s algorithm

Qualitative results using Piccinelli’s algorithm are shown in Fig. 1. Firstly, results of varying the resampling length *r* are shown in Fig. 1a, where we observe how the number of bends is increased from 5 to 15 as *r* decreases. Secondly, results of varying the smoothing factor $\lambda$ are shown in Fig. 1b, where the two leftmost models have 8 and 7 bends, respectively, in contrast to 5 for the rightmost model. Finally, results of varying the number of iterations *N* are shown in Fig. 1c, where an additional 6th bend is detected for $N=50$, whereas an increase of iterations show no noticeable difference.

**Fig. 2:**
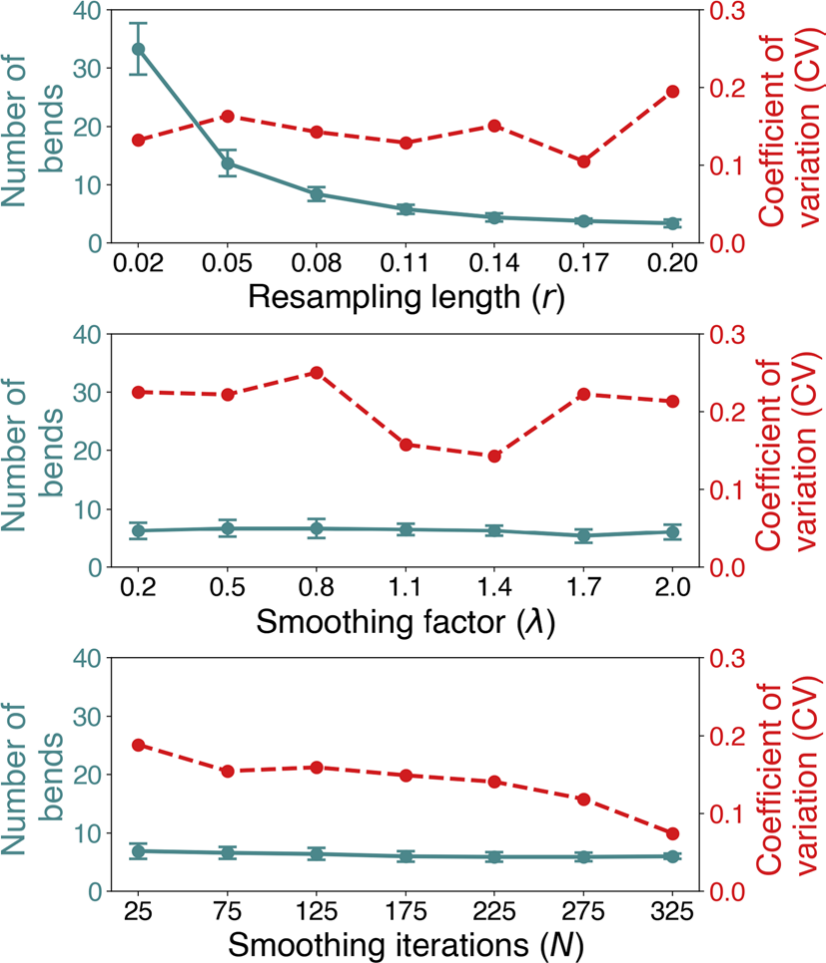
Plot of the mean number of detected bends and standard deviation, and their respective *CV*, versus the variable parameter, computed from 10 models using Piccinelli’s algorithm. Values are homogenized across all subplots

Quantitative results are presented in Fig. 2a, where we observe that the number of detected bends behaves inversely proportional to *r*, with a mean of 33 bends detected for $r=0.02$, in contrast to 3 bends for $r = 0.2$. The results of varying $\lambda$ are shown in Fig. 2b, where we observe a slight increase, followed by a decrease in the number of bends as the magnitude increases, varying around 6 bends. Finally, the results of varying *N* are shown in Fig. 2c, showing a steady decrease in detected bends as *N* increases, transitioning from a mean of 7 to 6 bends.

#### Bogunović’s algorithm

**Fig. 3:**
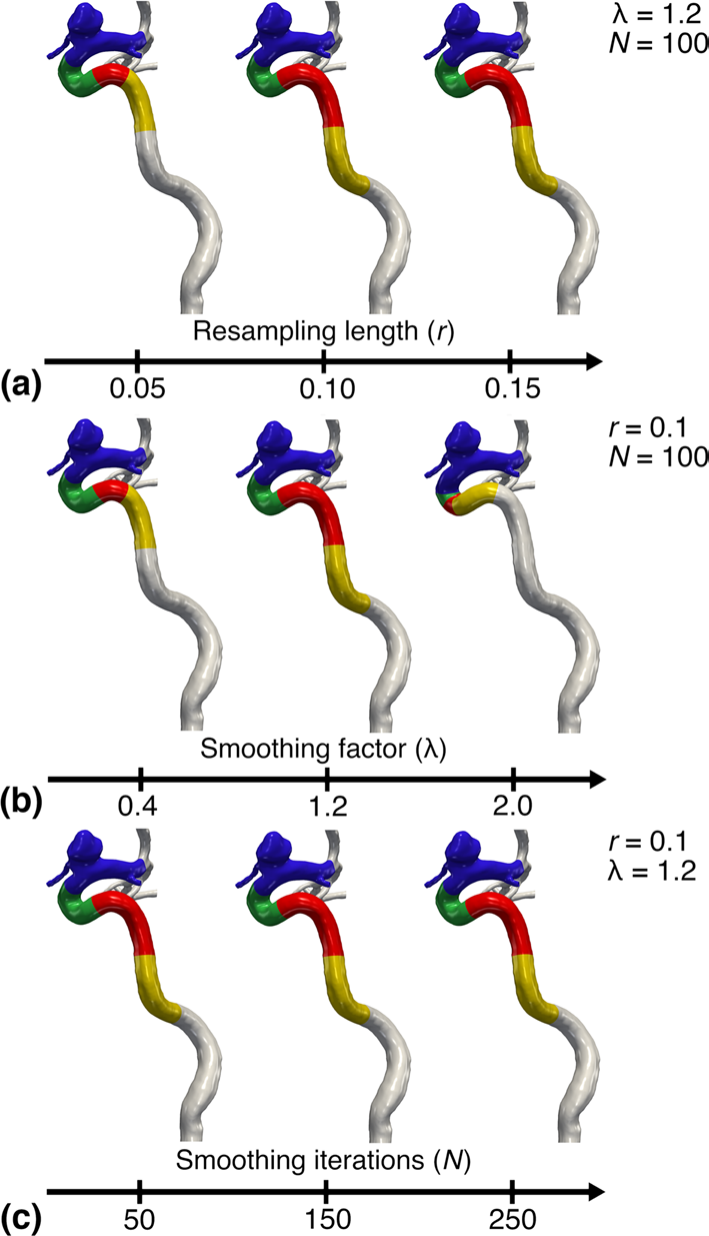
Results using the algorithm by Bogunović et al., where we have varied the three main input parameters. For row **a**,**b**, and **c** we have varied *r*, $\lambda$, and *N*, respectively, for a representative model

Qualitative results using Bogunović’s algorithm are shown in Fig. 3. Firstly, results of varying r are presented in Fig. 3a, where the leftmost model harbors an evidently short (red) bend, while the remaining models show no noticeable differences. Secondly, results of varying $\lambda$ are shown in Fig. 3b, where the left- and rightmost model contain particularly short bends, in contrast to the default model in the middle. Lastly, results of varying *N* are presented in Fig. 3c, where there are no noticeable differences, and the bends are adequately detected, regardless of *N*.

**Fig. 4:**
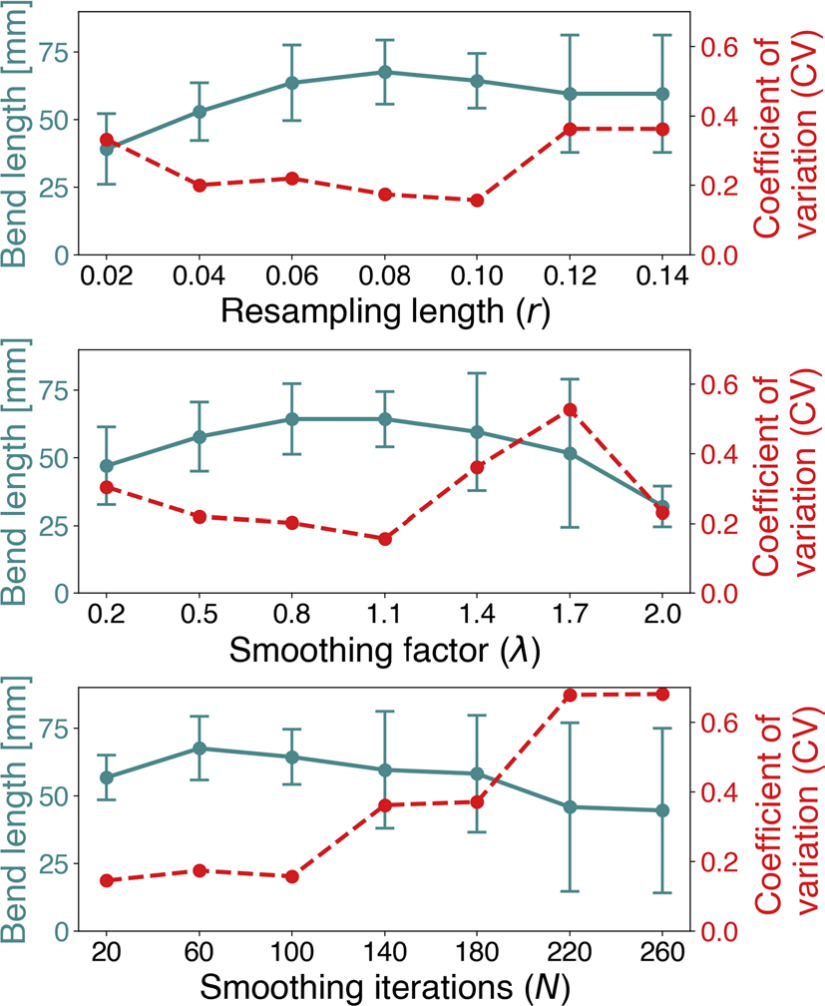
Plot of the mean total bend length and standard deviation, and the respective *CV*, versus the variable parameter, computed from 10 models using Bogunović’s algorithm. Values are homogenized across all subplots

Quantitative results are presented in Fig. 4. The results of varying *r* are presented in Fig. 4a, where we observe a steady growth in bend length towards $r=0.08$, while further increase contributes little to no value. The plot also shows how the lowest deviation appears for $r=0.1$, reflected by the *CV*, followed by a rapid increase as *r* increases. Low ($\lambda =0.2$) and high ($\lambda =2.0$) smoothing factors result in a shorter portion of the ICA being landmarked, shown in Fig. 4b. In contrast, around $\lambda =1.1$, the length averages at 60 mm, and harbors a minimum *CV*. When varying *N*, we observe a steady increase in deviation and decrease in length as $N > 100$, shown in Fig. 4c. In contrast, for $N \le 100$, the bends deviate much less and average around 60 mm in length. The lowest *CV* is attained at $N=20$, although for $N=60$ and $N=100$ the *CV* is similar.

**Fig. 5:**
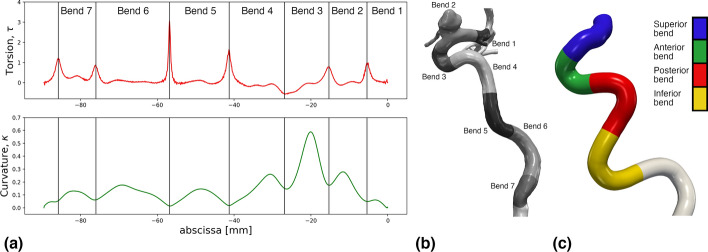
In **a**, the torsion and curvature profiles corresponding to surface model **b** are shown, including the seven interfaces. These define seven bends, shown on the model in **b**, and labeled in a proximal to distal direction. In **c**, the subdivision of the ICA as proposed by Bogunović et al. Here, the ICA is subdivided into four bends, and white areas denote the region outside of interest

### Verification and comparison of the landmarking algorithms

#### Verification

In Fig. 5a and b, we have applied our implementation to reproduce the original results by Piccinelli et al., specifically Fig. 4 in [12]. Similarly, in Fig. 5c, we reproduce the first landmarking result by Bogunović et al., shown in Fig. 5 in [19]. Albeit using only one model^1^, the correspondence between our and the original results suggests a successful implementation of the algorithm.

**Fig. 6:**
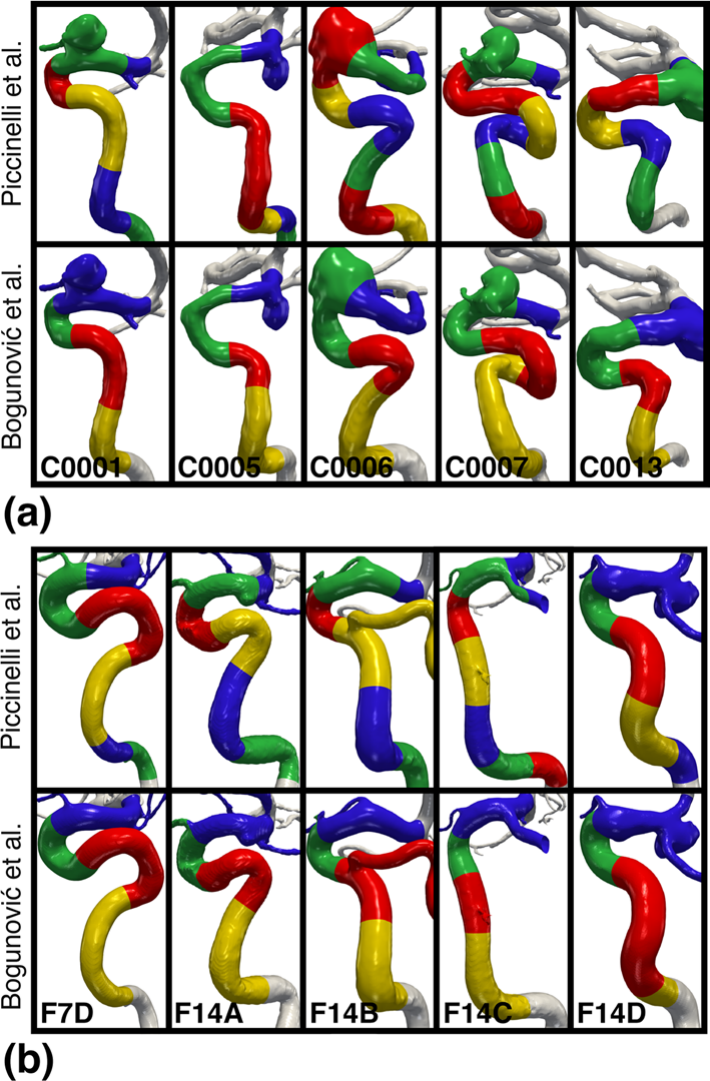
Application of the landmarking algorithms by Piccinelli et al. and Bogunović et al., applied to the Aneurisk cohort in **a**, and to the models provided by H. Bogunović in **b**. For both cohorts, we have landmarked the first and second rows using the algorithms by Piccinelli et al. and Bogunović et al., respectively. For the Aneurisk cohort, we have included the case name, and for the models provided by H. Bogunović, we have denoted each model their corresponding figure label in [19], e.g., **F7D** corresponds to Figure 7d

#### Comparison

Qualitative results are presented in Fig. 6, where Fig. 6a and 6b are Aneurisk models, and models provided by H. Bogunović are landmarked, respectively. The results of applying Piccinelli’s and Bogunović’s algorithm are shown in the top and bottom row, respectively.

Comparing the upper and lower row of Fig. 6a, the number of bends is arguably the biggest difference, inherently so by the algorithm design. Firstly, Piccinelli’s algorithm detects between six and eight bends, while Bogunović’s algorithm consistently captures four. Secondly, the first (blue) bend using Bogunović’s algorithm is detected as two bends in cases C0001, C0006, and C0013 with Piccinelli’s algorithm. Thirdly, for cases C0006 and C0007 the fourth (yellow) bend for Bogunović’s algorithm is captured as two bends (green and red) in the upper row. Additional results were presented for and acknowledged by Dr. Bogunović.^2^ In addition to the landmarking results presented in the second row of Fig. 6a, we performed an additional landmarking of 7 models from Aneurisk not shown here. The results were presented for Dr. Bogunović for verification, and he acknowledged that 10 out of 12 models were well landmarked. Two models had sub-optimal detection of the inferior (yellow) bend. However, the 2D projection of the models may have influenced the interpretation of the results.

In Fig. 6b, the models in the lower row can be qualitatively compared against results in [19], including the incorrect detection of the posterior (red) and inferior (yellow) bend in model F14D, as originally pointed out by Bogunović et al. Comparing the upper row models with the lower row, the largest differences are harbored in the middle three models. The superior (blue) bend is captured as two bends with Piccinelli’s algorithm, and captures the inferior (yellow) bend as two short bends for case F14C. In contrast, F14D is detected as five bends with Piccinelli’s algorithm and provides a better landmarking.

**Fig. 7:**
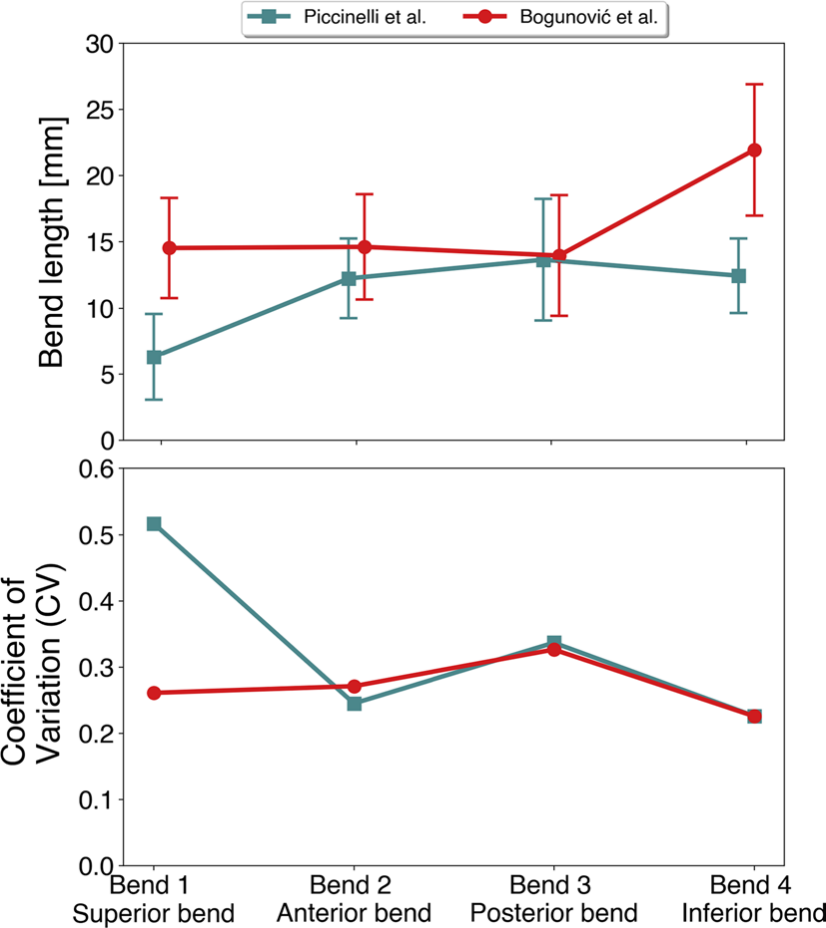
In **a**, a plot of mean length and standard deviation of the first four bends starting at the most proximal bend, traversing distally, for the models in Figure 6. In **b**, we have plotted the *CV*, per bend. We have excluded case F14D due to the incorrect landmarking with Bogunović’s algorithm

Quantitative results are presented in Fig. 7, but omitting the incorrectly landmarked model. The mean bend length and standard deviation is presented in Fig. 7a, which shows largest differences between the algorithms for the superior and inferior bend, respectively. The two bends are close to twice the length using Bogunović’s algorithm. In Fig. 7b, we show the *CV* for the respective bends, as defined in Equation 7. The results emphasizes the large relative deviance for the superior bend, where Piccinelli’s algorithm produces a high variability with a *CV* of 58%. In contrast, Bogunović’s algorithm has the highest variability for the posterior bend at 33%, although the overall variability is low.

**Fig. 8:**
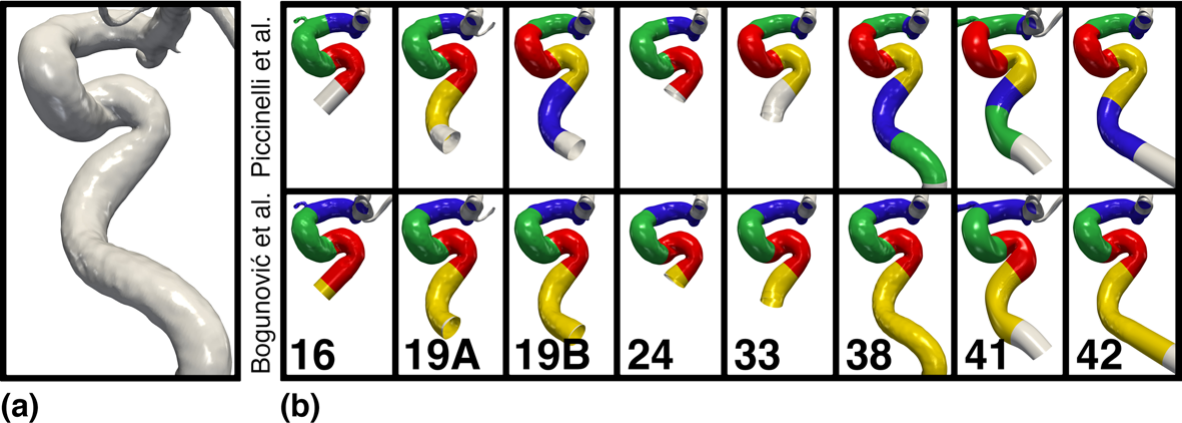
In **a**, the contour of case 5 of the 2015 CFD challenge, and in **b**, eight different segmentations based on the medical image. The model has been subdivided into separate bends using the two landmarking algorithms. The codes correspond to the team number in the 2015 CFD challenge, whereas team 19 submitted two different segmentations, marked as A and B, respectively

### Robustness of the landmarking algorithms

Qualitative results are shown in Fig. 8, where the alphanumerical characters are reflective of the respective participating teams of the former challenge. First, in the upper row of Fig. 8b, we have applied Piccinelli’s algorithm. Overall, the algorithm detects anywhere from three to six bends. Starting at the first (blue) bend and comparing horizontally, there is slight variability to how far upstream the next interface is detected. Consequently, the second (green) bend has large variability in location and length. The third (red) bend is located either as the anterior or posterior bend. Six of eight models detect a fourth (yellow) bend, detected as the posterior bend for all but one model (segmentation 19A). The only bend with a slight resemblance across all cases is the posterior bend, although it is captured as either the third (red) or fourth (yellow) bend. Therefore, allowing some leeway, there is a slight correspondence between bends in the cohort.

Second, in the lower row of Fig. 8b, we have applied Bogunović’s algorithm. Firstly, the superior (blue) bend is detected at indistinguishable location across all segmentations. Secondly, location and length of the anterior (green) bend is similar, with a tiny discrepancy in segmentations 24, 33, and 42, harboring a hardly noticeable, but slightly shorter bend. Thirdly, all cases appear to capture the same interface between the superior (blue) and anterior (red) bend. Finally, for seven of the eight cases, the posterior-inferior (red-yellow) interface coincides, with case 16 being the exception, which is influenced by the flow extension at the model inlet. In addition, the inferior (yellow) bend of segmentation 38 is arguably too long, harboring two curvature peaks.

**Fig. 9:**
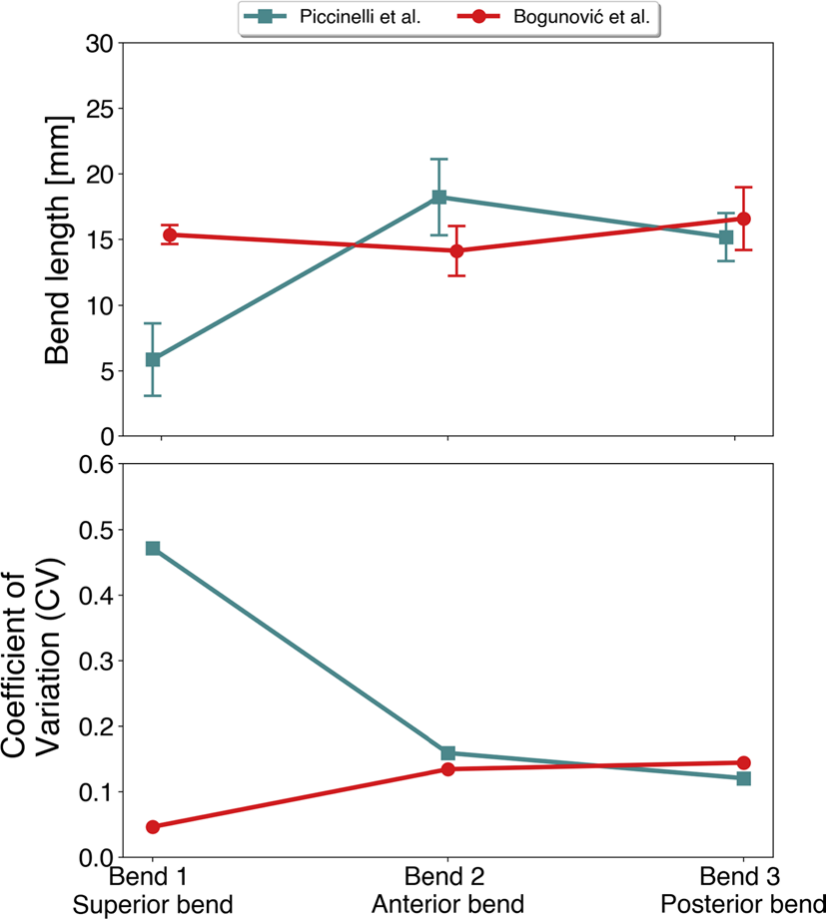
In **a**, a plot of mean length and standard deviation of the three first anatomical bends from Figure 8**b**. In **b**, we have plotted the *CV* for both algorithms

Quantitative results are shown in Fig. 9. The mean length and standard deviation for the three bends are presented in Fig. 9a, which highlights the particularly small deviation for the superior bend with Bogunović’s algorithm. For the remaining two bends, both algorithms perform well at capturing consistently sized bends. Overall, the bends detected with Piccinelli’s algorithms vary considerably more in mean length, compared to Bogunović’s algorithm. In Fig. 9b, we present the *CV* for the three bends. The results further support the variability observed in the qualitative results, with large differences in *CV* between the algorithms. Piccinelli’s algorithm shows high variability with a *CV* of 47% for the first bend. However, both the second and third bend lengths are consistent with a $CV < 20\%$. For Bogunović’s algorithm, the *CV* is generally low for all three bends, with a minimum of $CV<5\%$ for the superior bend.

## Discussion

**Fig. 10:**
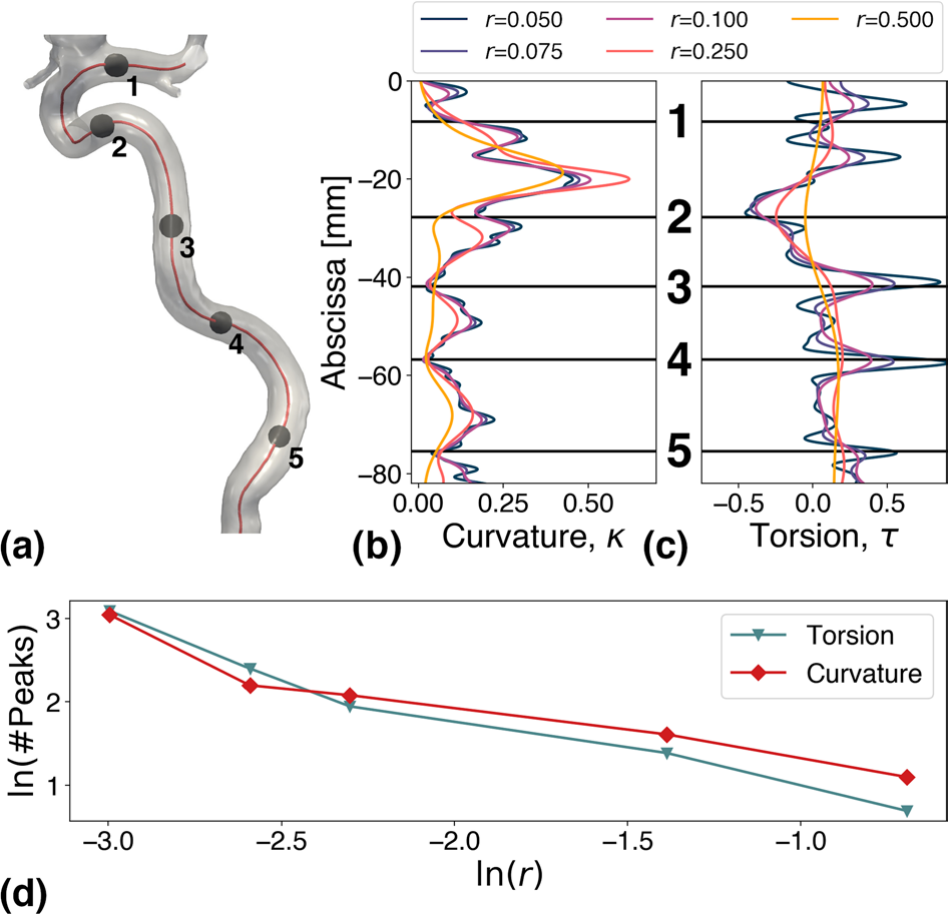
For the model in **a**, we show the effects of adjusting the resampling length (*r*) on the curvature **b** and torsion **c** profile. We have placed five manual landmarks in **a** between the major bends used as reference points in **b** and **c**. In **d**, the number of torsion and curvature peaks are plotted versus *r* in a log–log plot using the natural logarithm

### Sensitivity analysis

The two previous studies by Piccinelli et al. and Bogunović et al. have specified using Laplacian smoothing, but dedicated little attention to the centerline resolution, which is here shown to have the largest impact on the landmarking results. Therefore, to isolate and investigate the effects of centerline resolution on the geometric properties, and consequently on the landmarking results, we will be varying *r* for the representative model shown in Fig. 10a, keeping the remaining parameters fixed at $\lambda =1.2$ and $N=150$. The effects of varying *r* on the model’s curvature and torsion profile are shown in Figs. 10b, c, respectively, where values of $r < 0.1$ result in an increase in the number of saddle-like points located in the vicinity of the main peaks. Between $r=0.1$ and $r=0.25$, we observe a reduced number of extremum; whereas the peaks have completely diminished for $r=0.5$, represented by the orange curve. Figure 10d shows the overall impact *r* has on the number of peaks, which is considerably decreased as *r* is increased. Thus, values of $r > 0.1$ is shown to produce over-smoothed geometric profiles, following the centerline smoothing. In contrast, when $r < 0.1$ the geometric profiles are considerably noisy, creating an undesired amount of short bends regardless of the landmarking algorithm. Note that this analysis was performed for only one set of smoothing parameters. We therefore theorize that an adequate result might be obtained for different sets of centerline resolutions, using other smoothing parameters. Nonetheless, for centerlines representing models similar to the cohort used in this study, $r = 0.1$ is a reasonable choice.

Considering parameters retrospectively suggested by Dr. Piccinelli^3^, and judging by the sensitivity analysis, values of $\lambda \in [1.2, 1.5]$ appear to provide adequate results. Note that for $\lambda > 2.0$ Bogunović’s algorithm often fails to identify any bends.

Choices of *N* have shown consistent results for $N \in [20, 100]$, while much higher values will contribute to further smooth out the centerline, and consequently reduce the number of curvature and torsion peaks.

For parameters within these suggested ranges, Piccinelli’s algorithm is shown to produce an average of 6 to 7 bends. Furthermore, Bogunović’s algorithm consistently produces the four bends, at a total length ranging from 60 to 70 mm. This is in good correspondence with the clinical measurements by Vijaywargiya et al., who reported an average length of 68.9 mm for the ICA siphon [20].

### Verification and comparison of the landmarking algorithms

With the chosen set of parameters, we have verified our implementation of the landmarking algorithms. It should be noted that the verification was performed solely through qualitative analysis, and a more rigorous approach could include quantitative reference data if made available. Generally, software is verified through empirical analysis, and our implementation has shown to produce plausible results.

The comparison showed distinct differences between the algorithms, where Piccinelli’s algorithm repeatedly produced several bends, whereas Bogunović’s algorithm was less variable, consistently resulting in models with four bends, inherent in the algorithm design. As a result, we theorize that the two algorithms are not comparable in a meta-analysis. However, both work well for their purpose—capturing either geometrically defined bends or anatomical segments.

### Robustness of the landmarking algorithms

Piccinelli’s algorithm detects the ICA’s major bends at comparable locations, but without correspondence between bends due to large variability for the first (blue) bend. In contrast, Bogunović’s algorithm captures the first three bends at indistinguishable location and length, with an overall low variability.

The comparison shows that Piccinelli’s algorithm produces acceptable results, with similarly subdivided models, but is not robust enough to overcome the real-world inter-laboratory differences. In contrast, Bogunović’s algorithm is able to capture the underlying morphology irrespective of segmentation variability, although certain parameters are fitted to landmarking the ICA, thus at the cost of applicability to other vascular structures. Thus, for general tubular structures, we theorize that Piccinelli’s algorithm is sufficient at capturing bends, although it is prone to slight variability as shown here, while Bogunović’s algorithms is superior for carotid artery models.

### Limitations

Despite drawing authoritative conclusions in the previous sections, this work has some limitations. Firstly, the set of parameters determined through the sensitivity analyses produced adequate and robust results in our study, which can plausibly also be used in future applications. That being said, these parameter choices are not unique, and there may exist other parameter combinations that produce equally plausible or better results.

Secondly, the verification was based on a qualitative comparison that arguably is sub-optimal from a software engineering point of view. However, the software implementation of the previous studies was unavailable, and our open-source code produced adequate results.

Finally, the models used to study robustness were collected from a study with focus on aneurysm modeling pipeline consistency, and not accuracy of parent artery segmentation. Thus, the variability in segmentation may potentially be exaggerated, although these surface models were qualitatively similar to other studies [17].

### Implications and future considerations

Setting the limitations aside, this study has shown how the two algorithms perform adequately in isolation and fulfill the respective authors’ original purpose. It seems that Piccinelli’s algorithm is conceptually independent of application, and can be applied to any arbitrary tubular structure, but is somewhat vulnerable to model smoothness and noise. In contrast, Bogunović’s algorithm is robust and consistent, but with limited general applicability, as the threshold of the angles that separates each bend is specific to the ICA.

Having provided sufficient knowledge about the capabilities and limitations of both algorithms, combined with an open-source implementation, we would argue that the tools are readily available for others to use. The latter can potentially have a larger impact as many medical image-based studies are currently often limited by being operator-dependent, and labor-intensive. Combined, these factors are the bottlenecks that hinder large cohort studies that are needed to cover the vast variability in human anatomy needed for clinical impact [21].

The current results also highlight how input parameters can affect morphometric analysis results of the ICA. Like in many other scientific communities, it is actually fair to question “[...] whether we know what we think we know.” [22]. Therefore, it may currently not be excluded that meta-analyses of the ICA are confounded by the previously mentioned bottlenecks and subjectively chosen parameters. Finally, and maybe most importantly, there is absolutely no guarantee that any of the algorithms actually reflect anatomical landmarks commonly used in radiology. Addressing the latter is however beyond the scope of this paper, and would require development of new and more sophisticated algorithms in collaboration with neuroradiologists.

## Conclusion

This study provides an open-source implementation of the two existing algorithms for landmarking the ICA, and suggested parameters for models of similar morphology to the cohorts used in this study. Our investigation has also provided a comprehensive comparison of the algorithms, and shown their capability of capturing the vascular anatomy in the medical image irrespective of operator-dependent variability. Having provided in-depth knowledge about the capabilities and limitations of both algorithms combined with an open-source implementation, the tools are ready for others to use, although application to other vascular regions should be performed with caution.

**Fig. 11:**
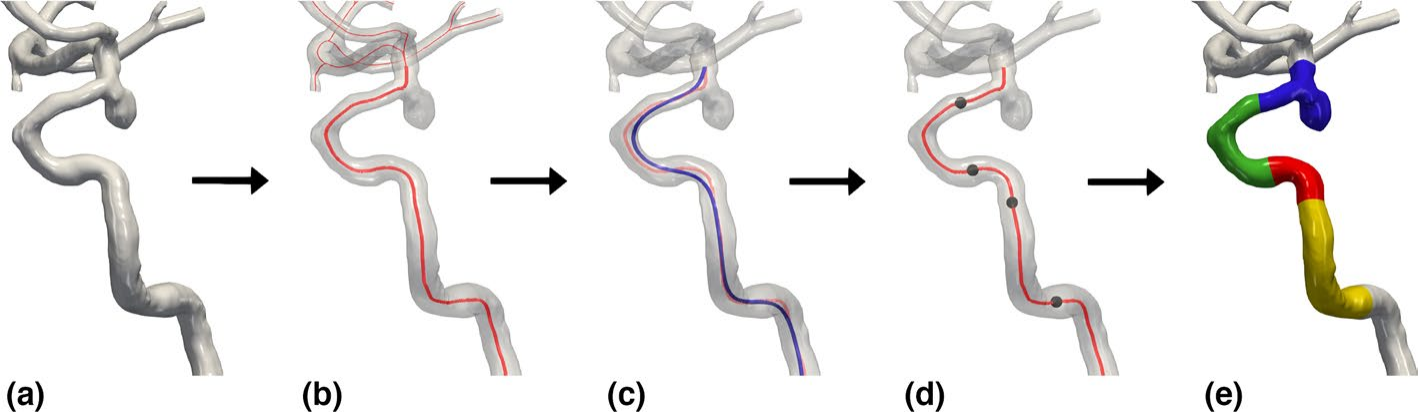
The general workflow for automated detection of bends in a tubular structure here applied to the ICA. We start with a 3D surface model (**a**), from which we compute the associated centerlines (**b**). A resampled and smoothed version of this line **(c)** is then used as input to the landmarking algorithms resulting in a set of interfaces (**d**), defining the anatomical bends **(e)**

## Methods

### Computation of the centerline

Landmarking the ICA was performed following the general workflow shown in Fig. 11. The starting point was a 3D model of the ICA, shown in Fig. 11a, from which the centerline was computed; see Fig. 11b. Following the methodology of previous work [12, 18], the centerline was here a proxy for computing geometric properties of the model and was parametrized as follows:

1 $$\begin{aligned} {\varvec{r}}(t) = (x(t), y(t), z(t)) \qquad t\in [0,1], \end{aligned}$$

where *t* is the arc length parameter.

**Fig. 12:**
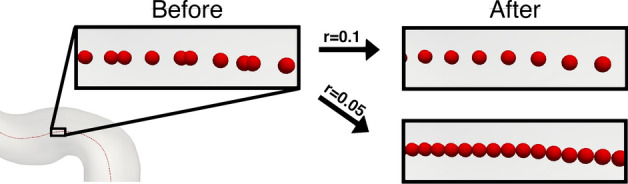
To the left, a portion of the centerline points computed directly from the Voronoi diagram. To the right, we have performed resampling of the centerline to achieve consistent spacing, here exemplified with $r=0.1$ and $r=0.05$

### Centerline resampling using a cardinal spline filter

The surface mesh resolution determines the density of the Voronoi diagram from which the centerline points are computed, resulting in non-uniform spacing [23]. For a fair comparison between the models, we resampled the centerline using a cardinal spline filter to achieve a consistent spacing *r* between the neighboring points, as shown in Fig. 12.

### Centerline smoothing using a Laplacian filter

Computation of geometric properties relies on numerical approximations of higher-order derivatives, which are sensitive to noise from the discrete centerline. As a remedy, we could either smooth the discrete curve, or the resulting geometric profile, where we chose the former approach, as shown in Fig. 11c. We applied a Laplacian filter to the centerline, which performed the following iteration *N* times:

2 $$\begin{aligned} {\varvec{r}}(t) \leftarrow {\varvec{r}}(t) + \lambda {\mathcal {L}}({\varvec{r}}(t)), \end{aligned}$$

where ${\mathcal {L}}(\cdot )$ is the discrete Laplace operator, and $\lambda$ is the smoothing factor.

To define a set of stable input parameters, we conducted a sensitivity analysis by varying *r*, *N*, and $\lambda$, as we were unaware of any specified values from previous studies.

### Computation of curvature and torsion

The curvature of the parametrized centerline from Equation 1, was computed as follows:

3 $$\begin{aligned} \kappa&= \frac{\Vert {\varvec{r}}'(t) \times {\varvec{r}}''(t)\Vert }{\Vert {\varvec{r}}'(t) \Vert ^3}, \end{aligned}$$

where the primes denote the derivatives with respect to *t*. Curvature is commonly interpreted to measure the deviation from a straight line. Geometrically, it is defined as the inverse of the radius of the osculating circle. Furthermore, following [24], torsion was computed as follows:

4 $$\begin{aligned} \tau&= \frac{{\varvec{r}}'''(t) \cdot [{\varvec{r}}'(t) \times {\varvec{r}}''(t)] }{\Vert {\varvec{r}}'(t) \times {\varvec{r}}(t) \Vert ^2 }. \end{aligned}$$

Torsion can be interpreted to measure how sharply the curve is twisting out of the osculating circle. Of notice, is that the highest derivative is of second and third order for curvature and torsion, respectively.

Alternatively, we may represent the curvature as a vector through the use of two frames: the Frenet–Serret frame and the parallel transport frame [25, 26]. In differential geometry, the Frenet–Serret formulas describe a particle’s kinematic properties moving along a curve in three dimensions. Generally, the formulas defining the Frenet–Serret frame are as follows:

5 $$\begin{aligned} \begin{bmatrix} {\varvec{T}}'(t) \\ {\varvec{N}}'(t) \\ {\varvec{B}}'(t) \\ \end{bmatrix} = \begin{bmatrix} 0 &{} \kappa &{} 0 \\ -\kappa &{} 0 &{} \tau \\ 0 &{} -\tau &{} 0 \end{bmatrix} \begin{bmatrix} {\varvec{T}}(t) \\ {\varvec{N}}(t) \\ {\varvec{B}}(t) \end{bmatrix}, \end{aligned}$$

where ${\varvec{T}}$, ${\varvec{N}}$, ${\varvec{B}}$ are the tangent, normal, and binormal vector, respectively. Of note is that ${\varvec{N}}$ points towards the center of the osculating circle, meaning we could define the curvature vector as ${\varvec{T}}'(t) = \kappa {\varvec{N}}(t)$, where $\kappa$ is the scalar curvature. Furthermore, the curvature vector could be expressed in the parallel transport frame, in combination with any orthonormal basis $\{{\varvec{E}}_1, {\varvec{E}}_2\}$, as follows:

6 $$\begin{aligned} {\varvec{T}}'(t) = k_1 (t) {\varvec{E}}_1(t) + k_2 (t) {\varvec{E}}_2(t). \end{aligned}$$

Here, $k_1$ and $k_2$ are the components of ${\varvec{T}}'(t)$ in the orthonormal basis. This representation of the curvature allowed for studying the torsion of a curve, but only computing the second derivative.

### The landmarking algorithms

The two landmarking algorithms are conceptually similar, both following the outlined workflow in Fig. 11. However, there are differences between step c and d in Fig. 11.

Piccinelli et al. identified locations of curvature and torsion extrema, and proceeded by defining a bend for each curvature peak enclosed by a proximal and a distal torsion peak. Bogunović et al. identified a set of anatomically inspired landmarks, and divided the vessel into a sequence of maximum four bends irrespective of the vessel length. By adopting the clinical nomenclature of Bogunović et al., the ICA was divided into the superior, anterior, posterior and inferior bend, colored blue, green, red, and yellow in Fig. 11e, respectively. Bogunović et al. defined bends as the curved parts of the centerline separated at a local curvature minimum. In contrast to Piccinelli et al., they expressed the curvature as a vector in the parallel transport frame, as defined in Equation 6. Hence, they detected bends by considering the trajectory of the curvature vector. As the curvature vector changes orientation, the basis vectors ${\varvec{E}}_1$ and ${\varvec{E}}_2$ remain stable along the curve, allowing for the measurement of the angle $\alpha$ between vectors. Using the curve representation in the $(k_1, k_2)$-space, four interfaces were detected from a proximal to distal direction with threshold angles of $\alpha = 45^{\circ }, 60^{\circ }, 45^{\circ },$ and $110^{\circ }$, respectively.

### Data acquisition and software

We have used three separate datasets to test various aspects of the algorithms, that have previously been described by Piccinelli et al. [12], Bogunović et al. [18], and Valen-Sendstad et al. [16].

Piccinelli et al. used a subset of 34 models from the open-source Aneurisk database, where surface models are segmented from 3D rotational angiography images [27]. Similarly, we selected the first five consecutive cases where the ICA exceeded 70 mm to include the entire ICA siphon. Thus, we restricted our selection to models hosting at least four major bends, to improve consistency of comparison by reducing the variability within the cohort. Details of the data acquisition and processing are explained further in [28].

Bogunović et al. performed segmentation of vascular models from 3D rotational angiographic images following the approach in [19], that was made available to the authors upon request. The models corresponded to the geometries shown in Figs. 5, 7, and 14 of [19], which allowed us to replicate the previously reported results for verification of the implementation. Hence, we applied the algorithms to six of the provided models, one for verification and the remaining five for comparison.

In consistency with the previous section, we chose the models that extended beyond the carotid siphon from the *2015 International Aneurysm Computational Fluid Dynamics (CFD) Challenge*, where 26 teams were provided medical images to study variability in segmentation and aneurysm hemodynamics [16, 29]. This resulted in 8 of 28 submitted models for assessing robustness.

We have included our implementation of the two landmarking algorithms in the open-source Python framework morphMan [30], which allows for manipulating morphological features in vascular geometries [31]. The morphMan framework is an extension of the vascular modeling toolkit (VMTK) [32], and inherits functionality such as computation of centerlines, curvature and torsion.

### Input parameter sensitivity analysis

To investigate the algorithms’ sensitivity to the three input parameters used to estimate curvature and torsion, we performed a qualitative analysis on a representative model from [27], followed by a quantitative analysis performed on a cohort of 10 ICA models. The 10 models were collected from [27] and [19] as described in Sect. .

For the qualitative results we varied $r=0.10 \pm 0.05$, $\lambda = 1.2 \pm 0.8$, and $N = 150 \pm 100$ consecutively, while keeping the others fixed. Piccinelli’s algorithm found an arbitrary number of bends, while Bogunović’s algorithm detected a maximum of four bends. Therefore, we computed the coefficient of variation for the number of bends, and bend length, respectively, defined as:

7 $$\begin{aligned} CV = \frac{\sigma }{\mu }, \end{aligned}$$

where $\mu$ is the mean and $\sigma$ is the standard deviation. The *CV* was used as a metric to measure the algorithm’s success, as we assumed there was a naturally low morphological variability in the ICA’s bends, supported by previous clinical morphometric studies [20, 33–35].

### Verification and comparison of the landmarking algorithms

We performed verification by comparing our results qualitatively against published results. Specifically, we reproduced Figure 4 in [12] and Figure 5 in [19], as they are the only visualizations of the respective algorithms.

Then, to see how the algorithms compared to each other, we performed a qualitative and quantitative comparison of the two algorithms using the same cohort of 10 models as in Sect. . Qualitatively, we considered the number of bends and their location. Quantitatively, we only included the four most proximal bends in our analysis to provide a fair comparison between the algorithms, and have presented the mean bend length with standard deviation, and their respective *CV*. For both verification and comparison, we set the input parameters to $r = 0.1$, $\lambda = 1.2$, and $N = 100$, based on the sensitivity analyses.

### Assessing robustness of the landmarking algorithms

To assess robustness, i.e., the ability to capture the underlying morphology irrespective of operator-dependent segmentation variability of medical images, we performed a case study where we applied both algorithms to eight different segmentations of the same medical image, collected from the 2015 CFD challenge. The landmarking results were compared qualitatively and quantitatively, similarly to our procedure in Sect. , with input parameters set to $r = 0.1$, $\lambda = 1.2$, and $N = 100$. For the quantitative comparison, we compared the anatomical bends between the two algorithms. Thus, with Piccinelli’s algorithm, the comparison against the anterior bend would vary, being detected as either the second or third bend. Similarly, the posterior bend was detected as either the third or forth anatomical bend. Provided identical medical images, and therefore zero physiological variability in the cohort, we considered low deviation in bend length as a measure of the algorithm’s robustness.

## Data Availability

All data, source code, and other materials used and generated for this article are publicly available: The source code for all components of this paper is licensed under GPL 3.0 and can be obtained from https://github.com/KVSlab/morphMan. *morphMan* is platform independent, written in the Python programming language. The dataset analyzed during the current study in Figs. 11, 1, 5b, and 6a is available in the Aneurisk repository, http://ecm2.mathcs.emory.edu/aneuriskweb/. The dataset analyzed during the current study in Figs. 3, 5**c**, and 6b are available from the corresponding author on reasonable request. The dataset analyzed during the current study in Fig. 8 are available on Figshare with the identifier https://doi.org/10.6084/m9.figshare.6383516.v2.

## Abbreviations

ICA: Internal carotid artery
CV: Coefficient of variation
CFD: Computational fluid dynamics

## Footnotes

1 We present an approximation of the surface model, with only the centerline available for this particular model. However, because the algorithm only relies on the centerline, we successfully landmarked the model.

2 In retrospect of the landmarking results presented in the second row of Figure 6a, we performed an additional landmarking of 7 models from Aneurisk not shown here. The results were presented for Hrvoje Bogunović for validation, and he acknowledged that 10 out of 12 models were landmarked adequately. Two models had sub-optimal detection of the inferior bend. However, the 2D projection of the models may have influenced the interpretation of the results.

3 Dr. Piccinelli provided suggested values for the relevant parameters, including $\lambda$ equal to 1.5 and 1.2 for computing curvature and torsion, respectively.
